# Factors contributing to disparities in mortality among patients with non–small‐cell lung cancer

**DOI:** 10.1002/cam4.1796

**Published:** 2018-09-28

**Authors:** Anish J. Mehta, Shannon Stock, Stacy W. Gray, David R. Nerenz, John Z. Ayanian, Nancy L. Keating

**Affiliations:** ^1^ Department of Medicine Brigham and Women's Hospital Boston Massachusetts; ^2^ Department of Mathematics and Computer Science College of the Holy Cross Worcester Massachusetts; ^3^ Department of Population Sciences City of Hope Cancer Center Duarte California; ^4^ Center for Health Policy and Health Services Research Henry Ford Health System Detroit Michigan; ^5^ Institute for Healthcare Policy and Innovation University of Michigan Ann Arbor Michigan; ^6^ Department of Health Care Policy Harvard Medical School Boston Massachusetts

**Keywords:** clinical cancer research, healthcare disparities, lung cancer, outcomes research

## Abstract

Historically, non–small‐cell lung cancer (NSCLC) patients who are non‐white, have low incomes, low educational attainment, and non‐private insurance have worse survival. We assessed whether differences in survival were attributable to sociodemographic factors, clinical characteristics at diagnosis, or treatments received. We surveyed a multiregional cohort of patients diagnosed with NSCLC from 2003 to 2005 and followed through 2012. We used Cox proportional hazard analyses to estimate the risk of death associated with race/ethnicity, annual income, educational attainment, and insurance status, unadjusted and sequentially adjusting for sociodemographic factors, clinical characteristics, and receipt of surgery, chemotherapy, and radiotherapy. Of 3250 patients, 64% were white, 16% black, 7% Hispanic, and 7% Asian; 36% of patients had incomes <$20 000/y; 23% had not completed high school; and 74% had non‐private insurance. In unadjusted analyses, black race, Hispanic ethnicity, income <$60 000/y, not attending college, and not having private insurance were all associated with an increased risk of mortality. Black‐white differences were not statistically significant after adjustment for sociodemographic factors, although patients with patients without a high school diploma and patients with incomes <$40 000/y continued to have an increased risk of mortality. Differences by educational attainment were not statistically significant after adjustment for clinical characteristics. Differences by income were not statistically significant after adjustment for clinical characteristics and treatments. Clinical characteristics and treatments received primarily contributed to mortality disparities by race/ethnicity and socioeconomic status in patients with NSCLC. Additional efforts are needed to assure timely diagnosis and use of effective treatment to lessen these disparities.

## 1 INTRODUCTION

Lung cancer is the leading cause of cancer‐related death in the United States. While comprising 14% of new cancer diagnoses, lung cancer accounts for 27% of cancer‐related deaths every year.^1^ Nearly 90% of lung cancer patients are diagnosed with non–small‐cell lung cancer (NSCLC), and most of these patients are diagnosed at advanced stages; only 21% survive more than 5 years.^2^

Numerous studies have documented disparities in survival among patients diagnosed with NSCLC. In particular, patients who are non‐white,^3^, ^4^, ^5^ low income,^6^, ^7^ insured by Medicaid,^8^ or uninsured^9^, ^10^, ^11^ have a higher mortality rate from NSCLC compared with other patients. However, it is unclear how clinical and treatment differences contribute to these survival differences. Racial/ethnic minorities are more likely than white patients to be diagnosed at advanced stages,^12^ have a greater number of medical comorbidities,^13^ and receive fewer appropriate treatments,^3^, ^14^, ^15^, ^16^, ^17^ all of which may contribute to increased mortality. Similarly, patients insured by Medicaid are diagnosed at a later stage and receive fewer appropriate treatments than patients with private insurance.^7^, ^18^ Lower income patients are less likely than higher income patients to receive curative treatments; they also have a poorer prognosis.^19^

Many of the studies examining survival disparities in patients with NSCLC are single‐center cohort studies that may not be generalizable or registry studies that lack individual‐level data. The Cancer Care Outcomes Research and Surveillance (CanCORS) study is a large multiregional observational study of newly diagnosed lung and colorectal cancer patients that offers the opportunity to avoid these shortcomings. Previous studies using this dataset have examined racial/ethnic differences in depressive symptoms,^20^ health‐related quality of life,^21^ and perceived an unmet need for supportive services^22^ among patients with lung cancer, but these have not examined differences in survival. In this study, we use the CanCORS dataset to better understand the factors contributing to survival differences in patients newly diagnosed with NSCLC.

## 2 METHODS

### 2.1 Data overview

Details of the CanCORS study design have been described elsewhere.^23^ Briefly, patients newly diagnosed with colorectal or lung cancer were enrolled at study sites across the country from 2003 to 2005. The sites include 5 geographically defined areas (Iowa, Alabama, and certain counties in California and North Carolina), 5 integrated healthcare systems, and 15 Veterans Affairs medical centers. Eligible patients were identified through rapid case ascertainment by cancer registries. Patients were interviewed by telephone a median of 20 weeks after their diagnosis and provided information about demographics, comorbidities, and treatments received. Further clinical information was abstracted from medical records (available for 80% of patients). Abbreviated and proxy versions of the survey were available to patients who were unable to complete a full survey or had died by the time of contact. The CanCORS study population has been shown to be representative of individuals in the United States who were diagnosed with lung or colorectal cancer.^24^ The study protocol was approved by the institutional review board at all participating institutions.

### 2.2 Study cohort

**Figure 1:**
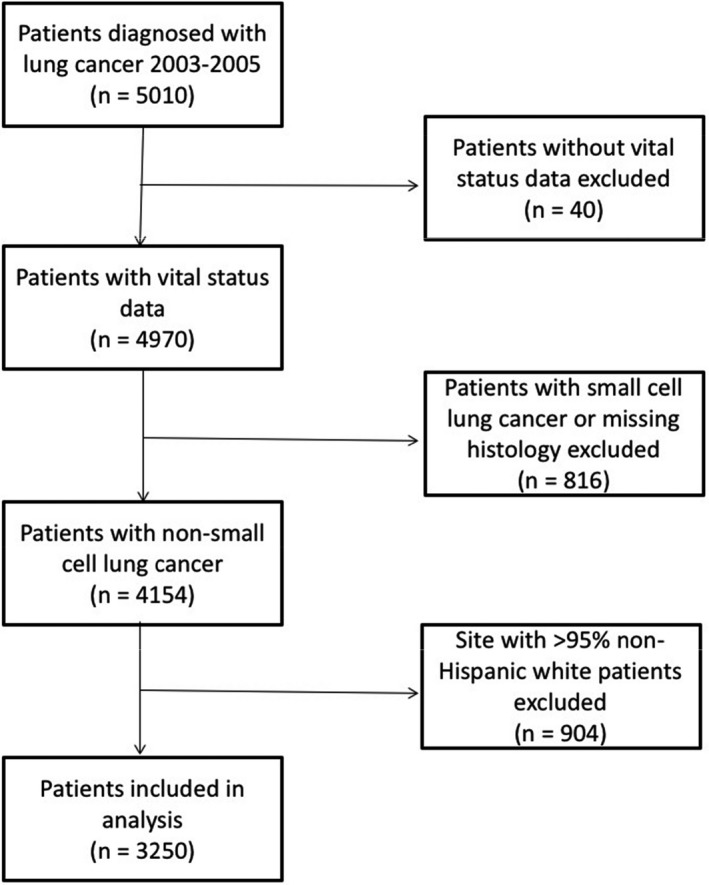
Flow chart of study participants

Among 5010 patients with lung cancer, we excluded 40 patients from one study site that did not ascertain survival and 816 patients with small‐cell lung cancer or missing histologic data. Because we assessed racial/ethnic differences, we also excluded 904 patients from one study site with >95% non‐Hispanic white patients, since that site would not inform such analyses. The final cohort included 3250 patients (Figure 1).

### 2.3 Variables

We grouped variables into three categories: sociodemographic characteristics, clinical characteristics, and treatments. Sociodemographic characteristics included the primary variables of interest, self‐reported race/ethnicity, annual household income, educational attainment, and insurance type, as well as sex, age (categorized into approximate quintiles), marital status, and enrollment in an integrated health system (including patients from the integrated healthcare system and Veterans Affairs study sites and patients in geographic sites enrolled in integrated health plans). Clinical characteristics included a number of self‐reported comorbidities,^25^ smoking status, and stage at diagnosis. Stage was assessed based on medical record abstractions or registry data when medical records were not available. Treatments included patient‐reported receipt of chemotherapy, surgery, or radiation. Variables were categorized as in Table 1.

**Table 1:** Characteristics and median survival of patients newly diagnosed with non–small‐cell lung cancer in CanCORS study 2003‐2005, *N* = 3250

	*N*	%	Median survival[Link] (days)	*P*‐value[Link]
Race/ethnicity and socioeconomic variables				
Race/ethnicity				
Non‐Hispanic white	2089	64	494	0.30
Non‐Hispanic black	512	16	476	
Hispanic	241	7	464	
Asian	226	7	526	
Other/multiracial	162	5	504	
Missing	20	1	548	
Income				
>$60 000	454	14	743	0.001
$40‐60 000	391	12	487	
$20‐40 000	726	23	391	
<$20 000	888	27	409	
Missing	791	24	611	
Education				
Some college or more	1182	36	583	0.001
High school diploma or vocational training	1253	39	459	
Non‐high school graduate	732	23	449	
Missing	83	2	361	
Insurance				
Private or HMO	832	26	620	0.001
Uninsured	46	1	290	
Medicaid, dual‐eligible, or VA/other government	474	15	441	
Medicare ± supplemental	1711	53	473	
Missing	187	5	446	
Sex				
Male	1851	57	405	0.001
Female	1399	43	653	
Age at diagnosis in years				
<60	744	23	570	0.001
60‐66	619	19	590	
67‐72	673	20	486	
73‐78	665	20	513	
>78	549	17	315	
Marital status				
Married/living with partner	1890	58	475	0.016
Divorced/separated/never married/widowed	1227	38	489	
Missing	133	4	874	
Integrated health system				
Integrated	1360	42	497	0.65
Non‐integrated	1890	58	490	
Clinical characteristics				
Stage at diagnosis				
Stage 1	818	26	2239	0.001
Stage 2	255	8	950	
Stage 3a/NOS	365	11	571	
Stage 3b	426	13	358	
Stage 4	1167	36	196	
Local/regional	54	1	290	
Missing	165	5	407	
Comorbidities				
0 comorbidities	703	22	475	0.001
1 comorbidity	833	26	497	
2 comorbidities	593	18	457	
3 comorbidities	332	10	391	
4 + comorbidities	312	10	336	
Missing	477	14	787	
Smoking status				
Never	306	9	587	0.001
Former	2193	67	393	
Current	246	8	705	
Missing	505	16	743	
Treatments received				
Radiation				
Yes	1208	37	341	0.001
No	1973	61	669	
Missing	69	2	501	
Chemotherapy				
Yes	1624	50	470	0.001
No	1553	48	574	
Missing	73	2	441	
Surgery				
Yes	1414	44	1489	0.001
No	1778	55	256	
Missing	58	1	537	

VA, veterans affairs; HMO, health maintenance organization; NOS, not otherwise specified.

Median survival from Kaplan‐Meier estimates.

Based on log‐rank tests.

Survival time was measured as days between a patient's diagnosis date and date of death; surviving patients were censored on the date last presumed to be alive. Date of death was obtained from four sources: CanCORS surveys (baseline or follow‐up, approximately 12 months after diagnosis), medical records, a Social Security Death Index match, or a National Death Index match. Queries on patient vital status from medical records or national death records were stopped at all sites by the end of 2012.

Missing data were infrequent (<6% of observations for all variables other than income [24%], smoking status [16%], and comorbidity [14%]). Missing data for all independent variables were imputed using multiple imputation.^26^ Vital status information was complete for all patients in the study population. Although some CanCORS studies have included information about social supports and baseline quality of life, such information was not collected by design for patients who were deceased at the time of the baseline survey and were not imputed; thus, those variables were not included in the analysis.

### 2.4 Statistical analysis

We calculated median survival time from diagnosis using Kaplan‐Meier estimates and compared differences using log‐rank tests. We used Cox proportional hazard models to assess the relative hazard of death. We tested for violations of the proportional hazards assumption by confirming that the log hazard‐ratio function for each covariate was constant over time. The stage at diagnosis variable interfered with the proportional hazards assumption due to 54 patients from one site coded as “local/regional.” In a sensitivity analysis excluding those 54 patients, the proportional hazards assumption was not violated, and the results did not differ (data not shown).

We first estimated the unadjusted hazard ratio for death based on race/ethnicity, income, education, and insurance type, as well as age, sex, smoking status, and receipt of care in an integrated health system. We then conducted three sequential models. The first model included all of these sociodemographic variables in a single model. The second model adjusted for sociodemographic and clinical characteristics at diagnosis, including stage, number of comorbidities, and smoking status. The third model adjusted for sociodemographic, clinical, and treatment variables, including receipt of surgery, chemotherapy, and/or radiation. Two‐sided *P*‐values <0.05 were considered statistically significant. All models adjusted standard errors by clustering for study site. Analyses were performed using Stata version 10 (StataCorp, College Station, TX).

## 3 RESULTS

Overall, 64% of patients were white, 16% black, 7% Hispanic, 7% Asian, and 5% other race/ethnicity. Among 2459 patients with income data, 27% of patients had incomes <$20 000/y. In total, 23% of patients had not completed high school, and 36% had two or more years of college. Only 1.4% of the population was uninsured at the time of diagnosis, 15% were insured by Medicaid, dually eligible, or insured by the VA, 53% of patients had Medicare, and 26% had private/health maintenance organization (HMO) insurance.

The median survival time was 470 days after diagnosis. Median survival by patient factors is presented in Table 1.

### 3.1 Survival differences

#### 3.1.1 Unadjusted analyses

In unadjusted analyses (Table 2), black patients had a slightly greater risk of death than white patients (HR = 1.05; 95% CI = 1.00‐1.10; *P* = 0.05). Hispanic patients tended to have a greater risk of death (HR = 1.08; 95% CI = 0.99‐1.19; *P* = 0.08); patients of Asian and other racial/ethnic descent did not differ from whites. Patients with lower incomes had higher mortality than those with incomes above $60 000 (HR = 1.34, 95% CI = 1.16‐1.55, *P* < 0.001 for incomes <$20 000; HR = 1.33, 95% CI = 1.18‐1.50, *P* < 0.001 for incomes between $20 000 and $40 000; and HR = 1.13; 95% CI = 1.04‐1.23; *P* = 0.005 for incomes between $40 000 and $60 000). Patients with less education also had higher mortality than patients with some college (HR = 1.29, 95% CI = 1.17‐1.42, *P* < 0.001 for patients who had not completed high school and HR = 1.13, 95% CI = 1.02‐1.25, *P* = 0.02 for high school graduates). Compared with patients who had private/HMO insurance, patients who were uninsured (HR = 1.39; 95% CI = 1.07‐1.80; *P* < 0.02), insured by Medicare (HR = 1.29; 95% CI = 1.11‐1.49; *P* = 0.001), or insured by Medicaid/VA/other government insurance (HR = 1.32; 95% CI = 1.05‐1.66; *P* < 0.02) had higher mortality. Mortality was lower for women versus men and for younger versus older patients (Table 2).

**Table 2:** Adjusted associations of characteristics with survival in patients newly diagnosed with non–small‐cell lung cancer

Variable	Unadjusted Association Hazard ratio (95% CI)	Model with Sociodemographic Variables Hazard ratio (95% CI)	Model with Sociodemographic and Clinical Variables Hazard ratio (95% CI)	Model with Sociodemographic, Clinical, and Treatment Variables Hazard ratio (95% CI)
Race/ethnicity and socioeconomic variables				
Race/ethnicity				
Non‐Hispanic white	1.00	1.00	1.00	1.00
Non‐Hispanic black	**1.05 (1.00‐1.10)**	1.00 (0.91‐1.09)	**0.87 (0.79‐0.97)**	**0.88 (0.80‐0.98)**
Hispanic	1.08 (0.99‐1.19)	1.07 (0.94‐1.21)	1.02 (0.89‐1.17)	0.97 (0.88‐1.08)
Asian	0.95 (0.82‐1.09)	0.97 (0.82‐1.14)	**0.83 (0.75‐0.92)**	**0.82 (0.73‐0.92)**
Other	0.92 (0.62‐1.35)	0.93 (0.66‐1.30)	0.95 (0.68‐1.31)	0.97 (0.74‐1.27)
Income				
>$60 000	1.00	1.00	1.00	1.00
$40‐60 000	**1.13 (1.04‐1.23)**	1.06 (0.98‐1.15)	1.04 (0.90‐1.20)	1.01 (0.87‐1.18)
$20‐40 000	**1.33 (1.18‐1.50)**	**1.20 (1.04‐1.38)**	**1.17 (1.00‐1.37)**	1.11 (0.93‐1.33)
<$20 000	**1.34 (1.16‐1.55)**	**1.17 (1.00‐1.36)**	1.18 (0.96‐1.46)	1.12 (0.92‐1.37)
Education				
Some college or more	1.00	1.00	1.00	1.00
High school diploma or vocational training	**1.13 (1.02‐1.25)**	1.09 (0.99‐1.20)	1.05 (0.90‐1.23)	1.04 (0.91‐1.18)
Non–high school graduate	**1.29 (1.17‐1.42)**	**1.16 (1.06‐1.26)**	1.09 (0.93‐1.28)	1.02 (0.87‐1.19)
Insurance				
Private insurance/HMO	1.00	1.00	1.00	1.00
Uninsured	**1.39 (1.07‐1.80)**	1.24 (0.92‐1.66)	1.26 (0.88‐1.80)	1.04 (0.68‐1.60)
Medicaid ± Medicare, VA/other govt	**1.32 (1.05‐1.66)**	1.08 (0.85‐1.36)	1.13 (0.90‐1.42)	1.11 (0.89‐1.37)
Medicare ± supplemental	**1.29 (1.11‐1.49)**	1.06 (0.92‐1.22)	1.12 (0.94‐1.33)	1.14 (0.94‐1.37)
Additional sociodemographic variables				
Sex				
Male	1.00	1.00	1.00	1.00
Female	**0.77 (0.70‐0.85)**	**0.77 (0.70‐0.83)**	**0.80 (0.77‐0.83)**	**0.79 (0.74‐0.84)**
Age at diagnosis				
<60	1.00	1.00	1.00	1.00
60‐66	0.96 (0.92‐1.01)	**0.92 (0.88‐0.97)**	1.01 (0.96‐1.06)	0.97 (0.89‐1.07)
67‐72	1.08 (0.99‐1.17)	1.00 (0.94‐1.07)	**1.17 (1.04‐1.30)**	1.08 (0.96‐1.22)
73‐78	**1.16 (1.01‐1.34)**	1.09 (0.99‐1.19)	**1.25 (1.06‐1.48)**	1.11 (0.92‐1.33)
>78	**1.56 (1.41‐1.73)**	**1.46 (1.31‐1.62)**	**1.69 (1.51‐1.90)**	**1.27 (1.07‐1.52)**
Marital status				
Married/living with partner	1.00	1.00	1.00	1.00
Divorced/separated/never married	1.02 (0.96‐1.08)	1.02 (0.96‐1.09)	1.02 (0.92‐1.13)	1.00 (0.90‐1.11)
Integrated health system				
Non–integrated	1.00	1.00	1.00	1.00
Integrated	1.02 (0.86‐1.21)	0.97 (0.80‐1.18)	0.95 (0.83‐1.08)	0.90 (0.81‐1.01)
Clinical characteristics				
Stage				
Stage 1	1.00		1.00	1.00
Stage 2	**1.81 (1.47‐2.23)**		**1.88 (1.54‐2.31)**	**1.98 (1.49‐2.64)**
Stage 3a/NOS	**2.46 (2.08‐2.91)**		**2.67 (2.34‐3.04)**	**2.34 (2.01‐2.73)**
Stage 3b	**3.39 (2.49‐4.61)**		**3.66 (2.87‐4.67)**	**3.05 (2.31‐4.03)**
Stage 4	**5.73 (4.49‐7.31)**		**6.35 (5.03‐8.03)**	**4.91 (3.60‐6.72)**
Local/regional	**2.78 (2.27‐3.40)**		**2.89 (2.24‐3.74)**	**2.27 (1.65‐3.11)**
Missing	**3.18 (2.04‐4.94)**		**3.45 (2.35‐5.07)**	**2.74 (1.79‐4.20)**
Comorbidities				
0 comorbidities	1.00		1.00	1.00
1 comorbidity	1.03 (0.92‐1.15)		1.09 (0.97‐1.24)	**1.12 (1.00‐1.25)**
2 comorbidities	1.06 (0.95‐1.18)		**1.11 (1.00‐1.22)**	**1.12 (1.02‐1.23)**
3 comorbidities	1.08 (0.89‐1.32)		1.11 (0.94‐1.31)	1.15 (0.99‐1.34)
4 + comorbidities	**1.32 (1.06‐1.65)**		**1.36 (1.08‐1.73)**	**1.32 (1.04‐1.67)**
Smoking status				
Never	1.00		1.00	1.00
Former	1.17 (0.98‐1.39)		**1.16 (1.07‐1.25)**	**1.12 (1.04‐1.23)**
Current	0.97 (0.80‐1.14)		0.96 (0.77‐1.21)	0.86 (0.61‐1.22)
Treatments received				
Radiation				
Yes	1.00			1.00
No	**0.64 (0.55‐0.75)**			0.90 (0.78‐1.04)
Chemotherapy				
Yes	1.00			1.00
No	0.88 (0.75‐1.03)			**1.65 (1.51‐1.81)**
Surgery				
Yes	1.00			1.00
No	**3.57 (3.14‐4.06)**			**2.21 (1.90‐2.58)**

VA, Veterans Affairs; HMO, health maintenance organization.

Bold values represent statistically significant associations (*P* < .05).

#### 3.1.2 Model 1—adjustment for sociodemographic variables

In the first model, we adjusted for sociodemographic characteristics (race, income, educational attainment, insurance type, sex, age, and marital status) and observed no difference in mortality by race/ethnicity or insurance type (Table 2). Adjusted mortality was higher for patients earning <$40 000 yearly compared with those earning >$60 000 yearly (HR = 1.20, 95% CI = 1.04‐1.38, *P* = 0.015 for those earning $20 000‐$40 000; HR = 1.17, 95% CI = 1.00‐1.36, *P* = .048 for those earning <$20 000). Patients with less educational attainment had higher mortality than those who attended college (HR = 1.16, 95% CI = 1.06‐1.26, *P* = 0.001 for those without a high school diploma; HR = 1.09, 95% CI = 0.99‐1.20, *P* = .065 for those with a high school diploma or vocational training).

#### 3.1.3 Model 2—adjustment for sociodemographic and clinical variables

In the second model, we adjusted for sociodemographic and clinical characteristics, including stage at diagnosis, number of comorbidities, and smoking status (Table 2). After this adjustment, black patients (HR: 0.87; 95% CI = 0.79‐0.97; *P* = 0.01) and Asian patients (HR: 0.83; 95% CI = 0.75‐0.92; *P* < .001) had lower mortality than non‐Hispanic white patients. Patients earning $40 000‐$60 000 had similar mortality as those earning >$60 000. Patients earning $20 000‐$40 000 had higher mortality than those earning >$60 000 (HR = 1.17; 95% CI = 1.00‐1.37; *P* = 0.049). The difference in mortality by educational attainment was no longer statistically significant.

#### 3.1.4 Model 3—adjustment for sociodemographic, clinical, and treatment variables

After adjustment for treatments received, including chemotherapy, surgery, or radiation, Asian and black patients continued to have lower mortality compared with non‐Hispanic white patients (Table 2). After this adjustment, there was no longer any difference in mortality by income. In this fully adjusted model, mortality was higher for men versus women, patients aged >78 versus <60, patients with advanced‐stage cancer, and patients with one or more comorbidities. Mortality was also higher for patients who did not undergo chemotherapy or surgery.

## 4 DISCUSSION

Consistent with previous studies examining disparities in care and outcomes for patients with NSCLC, we found that race/ethnicity, income, educational status, and insurance status were associated with higher mortality among patients with NSCLC. However, we found that these differences were no longer evident after adjusting for sociodemographic, clinical, and treatment variables.

In unadjusted analysis, black patients had a slightly higher risk of mortality compared with white patients, but the difference was small (median survival differences—18 days). This difference was notably smaller than the median differences by education (4.5 months), income (11 months), and insurance coverage (11 months), which likely represent clinically important disparities. The higher risk of mortality for black versus white patients was no longer evident after adjustment for other sociodemographic characteristics, including income, education, and insurance. In fact, after adjustment for differences in cancer stage and comorbidities, black patients had lower mortality than white patients. These findings differ from studies that show higher 5‐year mortality for black and Hispanic patients compared with non‐Hispanic white patients.^3^, ^4^, ^5^, ^27^ However, our population differs from these studies in that we have a multiregional cohort with a relatively small unadjusted difference in mortality between black and white patients, and we also had rich, self‐reported data about patients’ sociodemographic status. Other studies have documented that black patients with lung cancer have similar or better mortality than white patients after adjustment for sociodemographic factors^28^ or in settings with equal‐access health systems such as the military.^29^, ^30^ As a whole, this suggests that differences in access to treatment and timely diagnosis may play an important role in survival differences by race/ethnicity. Asian patients in this study had similar mortality as white patients in the unadjusted analysis, but had lower mortality than white patients after adjustment for clinical factors, including stage. We lacked data on molecular markers in our population; however, the better survival relative to white patients may reflect the higher frequency of favorable EGFR mutations in Asian patients with NSCLC.^31^

Patients earning below $60 000 per year had a 13‐34% higher risk of mortality than those earning more than $60 000. This difference was diminished in part by adjustment for more advanced stage at diagnosis and greater comorbidity burden among lower income individuals, and further diminished by adjustment for treatment. Previous studies have shown that low‐income patients are often diagnosed at a later stage than high‐income patients, and late‐stage disease is a strong predictor of poorer outcomes.^32^, ^33^ Patients who have low income may have worse outcomes for reasons not captured by stage at diagnosis or number of comorbidities. These may include severity of comorbidities, lack of social support, or even intensity of treatments received. Identifying strategies to ensure access to needed care in addition to early diagnosis will be important to addressing income‐related differences in care.

We also observed that patients with less education had 13‐29% increased risk of mortality than patients with more education. This difference was no longer evident after adjustment for clinical features at the time of diagnosis. Other studies have shown that lower educational attainment predicts poorer outcomes in patients with NSCLC.^34^ Our study extends such work by assessing patient‐reported educational attainment rather than imputing educational attainment by geocoded address data at an area‐ or census‐tract level. Our study also builds on prior work showing that patients with lower levels of education are diagnosed at later stages,^35^ a plausible mechanism leading to higher mortality.

The National Academy of Medicine defines health disparities as differences in care or outcomes that are not due to differences in clinical appropriateness or informed patient preferences.^36^ Although we had rich clinical data, we lacked information to allow us to fully understand whether the differences that persisted after adjustment for stage at diagnosis, comorbidities, and treatments received were related to differences in access and receipt of high‐quality care or other factors like patient preferences and clinical appropriateness. However, given the magnitude of survival differences demonstrated in the unadjusted analysis, these likely represent clinically relevant disparities that are explained by clinical and treatment‐related factors.

Our study's strengths include its diverse and multiregional patient population from across the United States as well as the rich clinical data from patients, medical records, and cancer registries. However, our study has several limitations. First, patients were diagnosed more than a decade ago, and we cannot be certain that findings would be similar in current cohorts, although other evidence suggests that racial/ethnic and sociodemographic disparities remain a problem for patients diagnosed with lung cancer.^27^, ^37^, ^38^, ^39^ Second, our patient population had a lower rate of uninsurance than the general US population. This may reflect the geographic regions we studied. It is also possible that patients without insurance were less likely to enroll in the study, although we recruited patients primarily from population‐based registries, and our study cohort was representative of patients in these areas with newly diagnosed cancers.^24^ Lastly, we ascertained treatments based on patient report, although other evidence suggests that patients can accurately report major cancer treatments such as surgery, radiation, and chemotherapy.^40^, ^41^

In conclusion, we observed higher mortality for NSCLC for black versus white patients, patients with non‐private insurance, and patient with lower levels of educational attainment and income. The difference between black and white patients in survival was no longer evident after adjustment for sociodemographic factors, and black patients had better outcomes than white patients after adjustment for clinical characteristics and treatments received. Clinical characteristics at the time of diagnosis contributed to higher mortality for patients with less education and income. Different treatments received also contributed to higher mortality for low‐income individuals. Our study suggests that disparities in NSCLC mortality may be diminished with efforts to ensure early diagnosis and effective treatments.
